# A cfDNA fragmentomics-based liquid biopsy assay for early detection of oral cancer

**DOI:** 10.1186/s12885-026-16606-x

**Published:** 2026-08-15

**Authors:** Weiwei Wang, Tao Ding, Cuicui Liu, Xinyue Hong, Peng He, Wei Yang, Maoxi Zhong, Yuan Jiang

**Affiliations:** 1 Hunan Cancer Hospital, The Affiliated Cancer Hospital of Xiangya School of Medicine, Central South University, Changsha, Hunan 410013 China; 2 Geneseeq Research Institute, Nanjing Geneseeq Technology Inc., Nanjing, Jiangsu 210032 China; 3 Department of Cancer Center, Chongqing University Three Gorges Hospital, Chongqing, No. 165 Xincheng Road, Wanzhou District, Chongqing 404000 China; 4 Clinical Laboratory of Hunan Cancer Hospital, Affiliated Cancer Hospital of Xiangya School of Medicine, Central South University, No. 283 Tongzipo Road, Yuelu District, Changsha, Hunan 410013 China

**Keywords:** Oral cancer, Early detection, Cell-free DNA, Fragmentomics, Low depth whole genome sequencing

## Abstract

**Background:**

Early detection of oral cancer improves outcomes, but remains limited by the invasiveness and low sensitivity of current screening methods.

**Methods:**

This study included 342 participants (200 in the training and 142 in the validation cohorts). Plasma cfDNA underwent low-depth whole-genome sequencing, with data normalized to a standardized 5× coverage via down-sampling to ensure consistent analysis. Three cfDNA-derived features, including fragment size ratio (FSR), copy number variation (CNV), and repetitive element profiles (REP), were extracted and integrated using a stacked machine learning ensemble to generate prediction scores. Model performance was evaluated by area under the receiver operating characteristic curve (AUC), sensitivity, and specificity with 95% confidence intervals.

**Results:**

In the training cohort, oral cancer patients had a median age of 53 years (91% male), while healthy individuals had a median age of 57 years (37% male). In the validation cohort, the median ages were 53 and 57 years, with male proportions of 83.1% and 38.0% in the cancer and healthy groups, respectively. Cancer samples exhibited shorter cfDNA fragments, recurrent CNV gains at 3q and 8q, and losses at 3p and 5q. The individual AUCs for FSR, CNV, and REP were 0.973, 0.983, and 0.979 in the training cohort, and 0.960, 0.982, and 0.972 in the validation cohort, respectively. The integrated stacked model achieved AUCs of 0.996 (95% CI: 0.991-1.000) and 0.988 (95% CI: 0.975–0.988) in the training and validation cohorts, respectively. To prioritize high specificity for large-scale screening, a decision threshold was established in the training cohort to target 98.0% specificity, which yielded a corresponding sensitivity of 98.0%. When this pre-fixed threshold was blindly applied to the independent validation cohort, a sensitivity of 94.4% and a specificity of 95.8% were achieved. Prediction scores were not significantly associated with age, sex, or tumor site.

**Conclusions:**

The cfDNA fragmentomics-based stacked model distinguished oral cancer from healthy individuals, including early stage disease, supporting its potential for noninvasive approach for oral cancer detection.

**Supplementary Information:**

The online version contains supplementary material available at https://doi.org/10.1186/s12885-026-16606-x.

## Introduction

Oral cancer is a malignant tumor originating from the tissues of the mouth, including lips, tongue, buccal mucosa, gingiva, and the floor of the mouth. More than 90% of oral cancers are oral squamous cell carcinoma (OSCC) [1]. Early-stage oral cancer often presents with subtle or painless clinical signs, which frequently go unnoticed by patients, leading to a substantial proportion of cases being diagnosed at advanced stage. The overall 5-year survival rate of oral cancer is approximately 40%, whereas the rate exceeds 85% in early-stage cases [2], indicating the importance of early detection.

Traditional screening methods for oral cancer include visual inspection, palpation, vital staining techniques, oral cytology, and tissue biopsy, each with inherent limitations [3]. Visual examination and palpation depend heavily on the clinician’s experience, while vital staining has a high false-positive rate and risk of overdiagnosis. Oral cytology is insufficiently sensitive for detecting early-stage disease, and tissue biopsy, the diagnostic gold standard, is invasive and may cause discomfort, reducing patient compliance. Moreover, tumor heterogeneity across different oral sites further complicates screening. Therefore, more convenient and reliable methods for early oral cancer detection are urgently needed.

With the advances in molecular biology and genomics, liquid biopsy has emerged as a promising noninvasive tool for early cancer detection [4]. Common analytes include cell free DNA (cfDNA), cfRNA, exosomes and metabolites [5]. cfDNA mainly originates from apoptotic or necrotic cells, including both normal and tumor cells. In healthy individuals, cfDNA fragments peak at approximately 167 bp, whereas tumor-derived cfDNA (ctDNA) fragments tend to be shorter [6]. cfDNA carries both genetic and epigenetic information, making it a valuable biomarker for cancer detection.

Multiple cfDNA features, such as methylation and fragmentomics, have been applied in early cancer detection [7–9]. Fragmentomic features, including copy number variation (CNV), fragment size, coverage and end motifs, have demonstrated high diagnostic potential. In this study, we developed a cfDNA fragmentomics-based assay for early detection of oral cancer. Using a training cohort of 100 oral cancer patients and 100 healthy controls, plasma cfDNA was analyzed via low depth whole-genome sequencing (WGS). Three cfDNA features, including fragment size ratio (FSR), CNV, and repetitive element profiles (REP), were integrated using a stacked ensemble machine learning approach to construct a predictive model. The model was subsequently evaluated in an independent validation cohort comprising 71 oral cancer and 71 healthy individuals.

## Materials and methods

### Study population and clinical enrollment

**Fig. 1:**
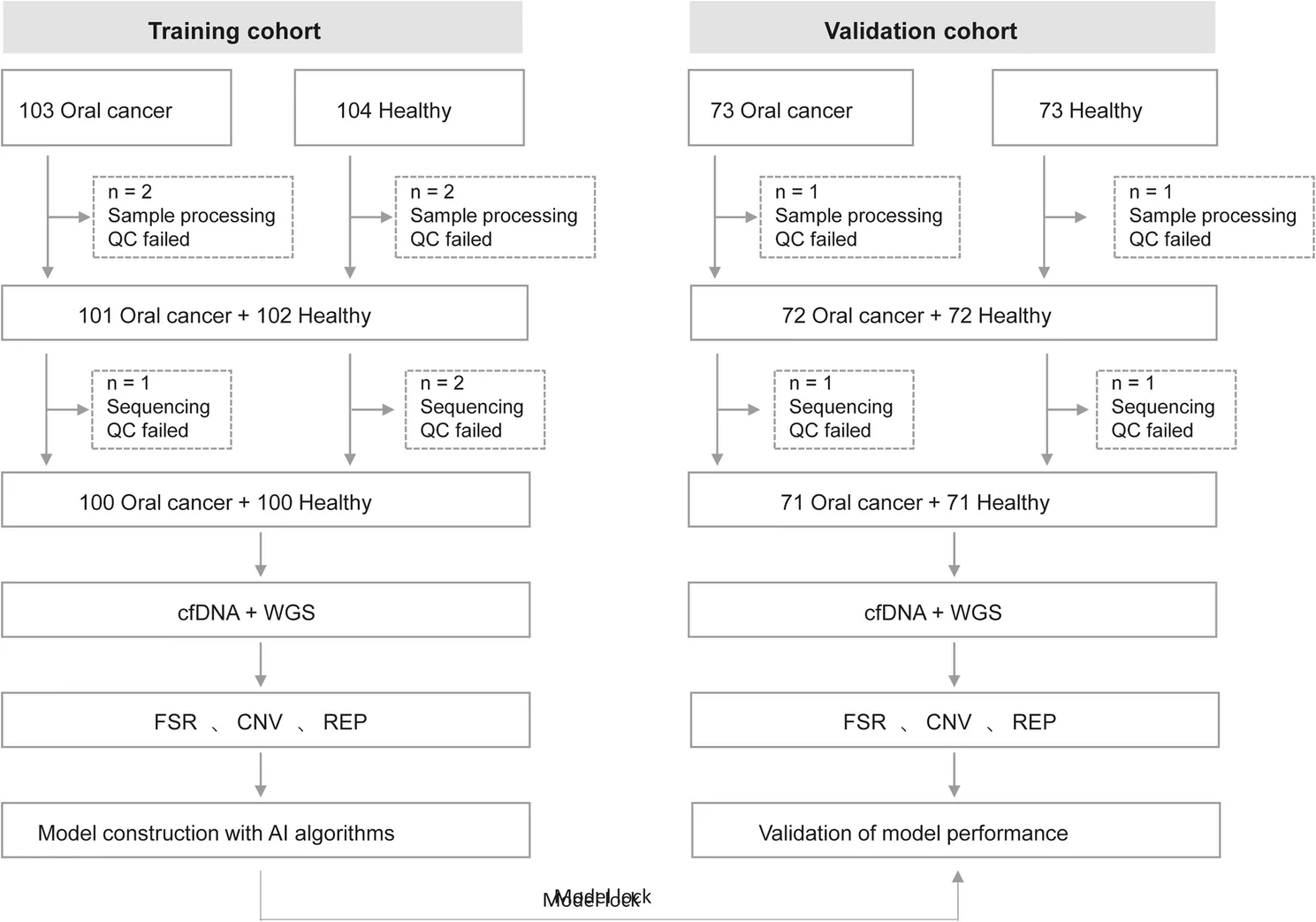
Study design and workflow. Following participants enrollment, cfDNA was isolated from blood samples and analyzed by low depth whole-genome sequencing (WGS). Samples that failed quality control were excluded. Three fragmentomic features, including fragment size ratio (FSR), copy number variation (CNV), and repetitive element profiles (REP), were extracted from sequencing data. A predictive model based on cfDNA features was constructed using a stacked machine learning ensemble in the training cohort and evaluated in the validation cohort

Participants in both training and validation cohorts were enrolled at a single clinical center (Hunan Cancer Hospital). The training cohort included patients pathologically confirmed with oral cancer and matched healthy controls enrolled chronologically between May and November 2024. The validation cohort was recruited prospectively over the subsequent six months (Fig. 1). To optimize machine learning training and ensure unbiased performance evaluation, we pre-specified a 1:1 case-to-control ratio for both cohorts. Anticipating sample attrition, we initially sequenced a larger pool of clinically eligible participants. Following strict pre-defined sequencing quality control (QC) filtering, we enforced the 1:1 design by selecting participants on a strictly chronological basis. The first 100 consecutive eligible OSCC cases and 100 matched controls (enrolled May–Nov 2024) that passed all QC metrics were assigned to the training cohort. The validation cohort (71 pairs) was chronologically formed from subsequent enrollments. Surplus samples that passed QC but exceeded the symmetric cohort targets were administratively excluded to maintain perfect class balance. Importantly, this chronological selection was finalized prior to fragmentomic feature extraction and model training, ensuring the process was completely blind to predictive outcomes.

Patients were identified through clinical referrals for suspicious oral lesions, such as persistent ulcers or mucosal changes. While early-stage cancers were often painless (physically asymptomatic), all cancer patients presented with visible clinical signs prompting biopsy. Histopathological diagnosis was performed by at least two senior oral pathologists according to the WHO Classification of Tumors (Head and Neck Tumors, 5th edition, 2022) [10]. Only cases of definitive OSCC with stromal invasion beyond the basement membrane were included. Any cases showing only intraepithelial neoplasia or premalignant lesions (e.g., simple leukoplakia) were strictly excluded. Healthy controls were apparently healthy volunteers recruited from the health examination center of Hunan Cancer Hospital during routine physical examinations, rather than patients receiving treatment for other diseases. At enrollment, all control participants underwent clinical oral examination, medical history review, and routine clinical/biochemical assessment. Individuals with a prior history of cancer, suspicious oral mucosal lesions, clinical or biochemical findings suggestive of malignancy, active severe inflammatory conditions, or severe systemic comorbidities were excluded. From the eligible pool, the first 100/71 consecutive cases and matched controls were retained to fulfill the pre-specified symmetric cohort sizes. All eligible participants were aged between 18 and 85 years, had no prior cancer history, and provided informed consent. This study followed the ethical principles of the Declaration of Helsinki and was approved by the Ethics Committee of Hunan Cancer Hospital (No. SBQLL-2022-273).

### Sample collection and cfDNA extraction

Approximately 10 mL of peripheral blood was collected from each participant using identical specialized tubes. To minimize pre-analytical bias and technical noise, a unified standard operating procedure (SOP) was enforced: all samples were transported at a constant 4 °C, and plasma was separated within 2 h of collection using an automated liquid handling platform. cfDNA extraction was completed within 72 h using the QIAamp Circulating Nucleic Acid Kit (Qiagen) stored at -80 °C for subsequent sequencing. Extracted cfDNA was quantified by Qubit dsDNA HS Assay Kit (Thermo Fisher Scientifc).

### Low depth whole genome sequencing and batch control

To mitigate temporal batch effects, a “collect-then-process” strategy was implemented, where all qualified samples were pooled into a single experimental batch. Automated library construction was then performed on a Hamilton Microlab STAR platform (Hamilton Company) using the KAPA Hyper Prep Kit (KAPA Biosystems), followed by qPCR-based quantification by the KAPA SYBR FAST qPCR Master Mix (KAPA Biosystems). Paired-end 150 bp sequencing was conducted on the DNBSEQ-T7 platform (MGI). While the initial sequencing target was 7–8× coverage per sample, the actual raw sequencing yields typically ranged from 4–6×. Following quality control with Trimmomatic [11], raw reads were aligned to the human reference genome (GRCh37/ hg19) using the Burrows–Wheeler Aligner (BWA) [12], and PCR duplicates were removed using Picard toolkit (https://broadinstitute.github.io/picard/). Finally, to minimize coverage-related biases, sequencing data were normalized to a standardized depth of 5× by down-sampling high-coverage libraries. Samples with raw coverage slightly below 5× (retaining at least 3.5×) were kept at their native depth to maximize data utility. Notably, statistical analysis confirmed that the sequencing depth after down-sampling remained comparable between the cancer and healthy groups (Figure S1).

### Feature extraction and profile analysis

Feature extraction was conducted based on three cfDNA-derived dimensions: FSR, CNV, and REP. For FSR analysis, the autosomal genome was divided into 5 Mb bins. Following the standards established by Mathios et al. [13], we calculated the ratio of short (100–150 bp) to long fragments (151–220 bp) fragments. To highlight relative genomic fluctuations and ensure cross-sample comparability, fragmentation profiles were normalized using a two-step procedure: first, profiles were centered by participant by subtracting the mean ratio across all autosomal bins (resulting in a mean of zero for each individual); second, each donor’s profile was further scaled by its genomic standard deviation (δ). This transformation is mathematically equivalent to a Z-score transformation, resulting in a final fragmentation distribution centered at zero with a unit variance of one across the genome.

CNV analysis was performed using ichorCNA (v0.3.2) across 1 Mb bins [14]. Briefly, the autosomal genome (chromosomes 1–22), excluding centromeric and low-mappability regions, was divided into 2,475 non-overlapping 1 Mb bins. The read depth of each bin was adjusted for GC content and normalized against the default panel of normals (PON) provided with the ichorCNA software. The log₂ ratio between the experimental and PON baseline values was then calculated and smoothed using the LOESS algorithm. In addition to bin-level profiling, the tumor fraction (TF), defined as the proportion of tumor-derived DNA relative to total cell-free DNA, as estimated for each sample using the hidden markov model (HMM) framework within ichorCNA. To benchmark the relative advantage of our multi-dimensional model, the diagnostic performance of using TF alone as a standalone classifier was independently evaluated in both the training and validation cohorts. While all 2,475 genome-wide bins were utilized as input features for machine learning, we also specifically examined the normalized read depths for five key oncogenic markers (*CCND1*,* MYC*, *CDKN2A*, *PTEN*, and *RB1*) to validate the biological relevance of the detected alterations. These genes were selected for analysis based on their established roles as key genomic drivers in OSCC, as documented in prior oncogenic studies [15–17]. Their gene-level values were determined by averaging the log₂ ratios of the bins overlapping their respective genomic coordinates (hg19).

For REP analysis, the method was adapted from Annapragada AV et al. [18]. A total of 1,287 repeat types were extracted from the RepeatMasker track in chm13 (T2T-CHM13v2.0), of which 1,266 containing at least one unique k-mer were retained, and 14 HSATII subfamilies were added as a supplement, resulting in 1,280 repeat types encompassing long interspersed nuclear elements (LINEs), short interspersed nuclear elements (SINEs), long terminal repeats (LTRs), transposable elements, and human satellite families. Sequencing reads were aligned to the hg19 genome, and read counts with a mapping quality (MAPQ) ≥ 30 were normalized.

### Machine learning and model development

A machine learning classifier was developed for oral cancer detection using the extracted cfDNA features. The stacked model was developed using the H2O AutoML (v3.42) framework [19], providing an empirical, data-driven environment for comparing multiple machine candidate learners. Candidate base learners included generalized linear model (GLM) [20, 21], extreme gradient boosting (XGBoost) [22], gradient boosting machines (GBM) [23], deep random forest (DRF) [24], extreme randomized trees (XRT) [24], and H2O deep learning [25, 26]. Although some of these algorithms belong to related families, they implement different learning strategies, regularization mechanisms and optimization engines. By retaining them as separate candidates, we allowed AutoML leaderboard to empirically determine the optimal combination of models based on the observed data. The H2O deep learning module was implemented as a fully connected multi-layer feed-forward neural network (multi-layer perceptron), trained via stochastic gradient descent with back-propagation. The specific architectural configurations and tuned hyperparameters for each algorithm are detailed in Table S1. To prevent overfitting, a regularized estimation procedure was applied to all algorithms. For GLM, we utilized Elastic Net (L1/L2) regularization (functionally equivalent to glmnet) with automated grid search for α and lambda_search for λ. For GBM, XGBoost, and H2O deep learning, a stochastic random grid search was performed over learning and architectural parameters, while DRF and XRT were trained using default H2O configurations (Table S1).

The model building followed a hierarchical integration strategy. For each specific fragmentomic feature type (FSR, CNV, and REP), candidate base models were trained and evaluated; the intermediate model yielding the highest area under the receiver operating characteristic curve was selected for each category. These three best-performing feature-specific models were then integrated into a final stacked ensemble [27, 28]. This stacked model generated a continuous predictive score ranging from 0 to 1, with higher values indicating a greater likelihood of oral cancer.

Model evaluation followed a two-stage scheme. First, 5-fold cross-validation was conducted within the training cohort to generate out-of-fold (OOF) predictions for each base learner. These unbiased OOF predictions served as inputs to train a stacked model, constructing the final stacked ensemble classifier. Second, the final model, including its optimal classification threshold (determined by prioritizing 98.0% specificity on the training set), was locked. This locked model was then blindly applied to the independent validation cohort for out-of-sample performance evaluation.

### Statistical analysis

Continuous variables were compared using the Wilcoxon rank-sum test. The Jonckheere-Terpstra test was employed to evaluate ordered trends in predictive scores across T-stages. Diagnostic performance metrics, including AUC, sensitivity, and specificity, were reported with 95% confidence intervals (CIs) calculated via the Wilson score method. Differences in AUCs between models were assessed using the paired DeLong’s test. Multi-variable logistic regression was used to adjust for potential demographic confounders such as age and sex. All statistical tests were two-tailed, with *P* < 0.05 considered significant.

## Results

### Study design and cohort characteristics

The study design is illustrated in Fig. 1. In the training cohort, WGS was performed on blood samples that passed quality control, and cfDNA-derived features were extracted for analysis. AI algorithms were applied to integrate cfDNA features for model development. The independent validation cohort was used to evaluate model performance. Cohort characteristics were summarized in Table 1. The training cohort included 200 participants, comprising equal numbers of oral cancer patients (*n* = 100) and healthy individuals (*n* = 100). The age of oral cancer patients ranged from 30 to 78 years (median, 53 years), while healthy individuals ranged from 44 to 74 (median, 57 years). Male proportion was significantly higher in the cancer group (91%, 91/100) compared with the healthy group (37%, 37/100, *P* < 0.0001), reflecting the known epidemiological prevalence of oral cancer. The proportions of patients at stages I-IV were 9.0% (9/100), 21.0% (21/100), 26.0% (26/100), and 44.0% (44/100), respectively. Histologically, 47% (47/100), 44.0% (4/100), and 9.0% (9/100) of tumors were well, moderately, and poorly differentiated, respectively. In the validation cohort, 142 participants were enrolled at the same site, including 71 healthy individuals and 71 patients with oral cancer. The proportion of male patients was significantly higher in the oral cancer group (83.1%, 59/71) than in the healthy group (38.0%, 27/71; *P* < 0.0001). The distribution of disease stages I-IV was 18.3% (13/71), 25.4% (18/71), 15.5% (11/71), and 40.8% (29/71), respectively. The proportions of well, moderately, and poorly differentiated tumors were 49.3% (35/71), 42.2% (30/71), and 8.5% (6/71), respectively (Table 1).

**Table 1:** Clinical characteristics of patients with oral cancer and healthy controls in the training and validation cohorts

	Training cohort		Validation cohort	
	Cancer(*n* = 100)	Healthy(*n* = 100)	Cancer(*n* = 71)	Healthy(*n* = 71)
***Sex***				
Female	9.0%(*n* = 9)	63.0%(*n* = 63)	16.9%(*n* = 12)	62.0%(*n* = 44)
Male	91.0%(*n* = 91)	37.0%(*n* = 37)	83.1%(*n* = 59)	38.0%(*n* = 27)
***Age***				
median [min, max]	53 [30, 78]	57 [44, 74]	53 [23, 78]	57 [45, 71]
***Stage***				
I	9.0%(*n* = 9)	/	18.3%(*n* = 13)	/
II	21.0%(*n* = 21)	/	25.4%(*n* = 18)	/
III	26.0%(*n* = 26)	/	15.5%(*n* = 11)	/
IV	44.0%(*n* = 44)	/	40.8%(*n* = 29)	/
***Differentiation***				
Well	47.0%(*n* = 47)	/	49.3%(*n* = 35)	/
Moderately	44.0%(*n* = 44)	/	42.2%(*n* = 30)	/
Poorly	9.0%(*n* = 9)	/	8.5%(*n* = 6)	/

### cfDNA fragmentomic profiles

**Fig. 2:**
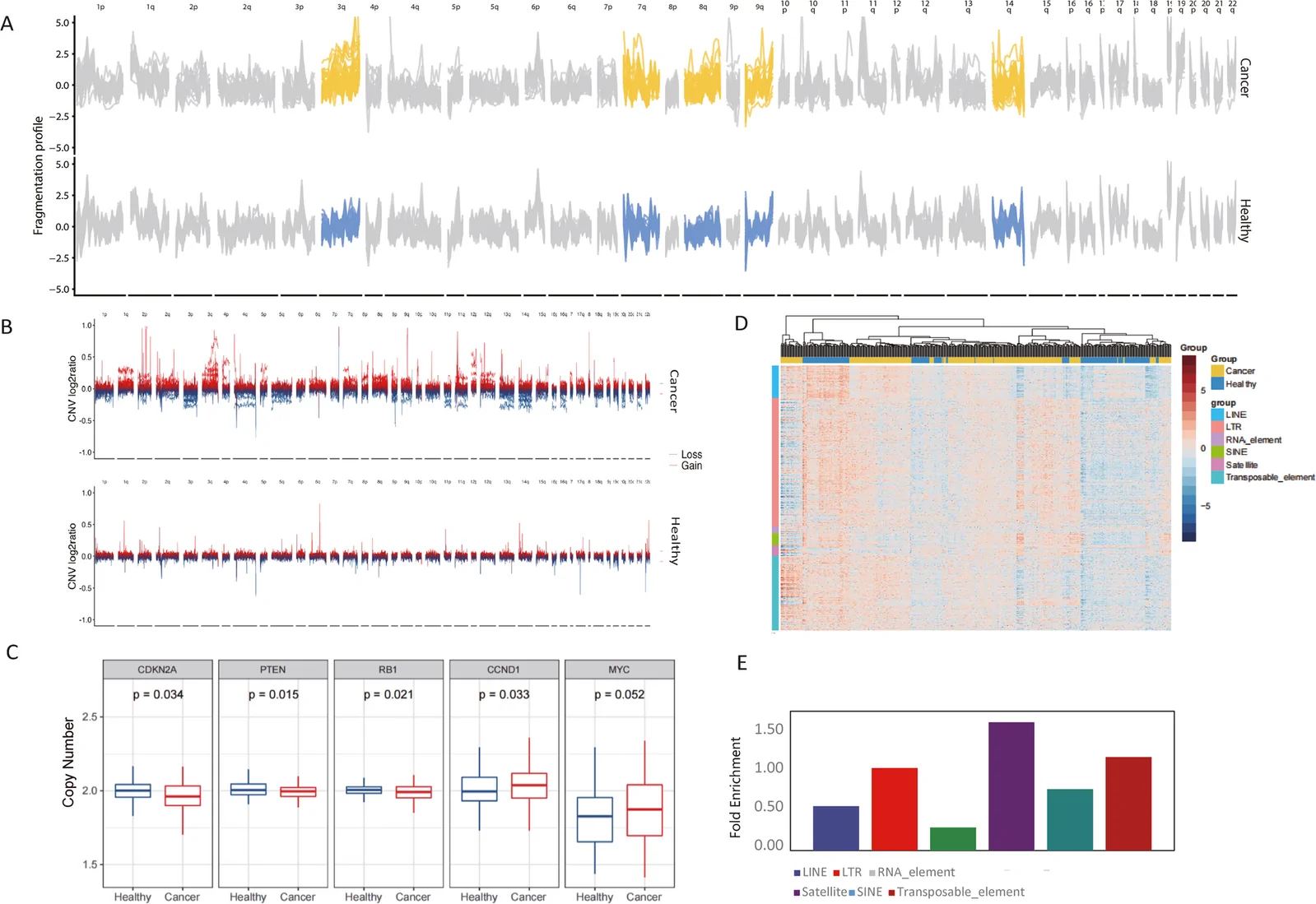
cfDNA features profiles in oral cancer and healthy samples. **A** Ratio of short fragments (100–150 bp) to long fragments (151–220 bp) in caner and healthy samples. **B** Copy number variation (CNV) profiles of cancer and healthy samples. **C** Gene-leve copy number alterations in cancer and healthy samples. **D** Repetitive element profiles in cancer and healthy samples. **E** Enrichment of repetitive elements in cancer and healthy samples. The y-axis for the FSR is scaled to [-5.0, 5.0] and the y-axis for the CNV log₂ratio is restricted to [− 1.0, 1.0] to optimize the visual resolution of recurrent genomic alterations by excluding extreme stochastic outliers

As shown in Fig. 2A, cancer samples exhibited a higher short-to-long fragment ratio, indicating a higher proportion of shorter fragments. CNV profiles revealed frequent gains at 3q and 8q, and losses at 3p and 5q (Fig. 2B). To evaluate the biological relevance of the detected alterations, we specifically assessed five established genomic drivers of OSCC pathogenesis. Significant copy number losses were detected in the tumor suppressor genes *CDKN2A* (*P* = 0.034), *PTEN* (*P* = 0.015), and *RB1* (*P* = 0.021) within the cancer group. In contrast, oncogenic drivers exhibited higher copy number values, with *CCND1* showing a significant gain (*P* = 0.033) and MYC displaying a numerical increase that approached statistical significance (*P* = 0.052; Fig. 2C). The repetitive element (REP) profiles were analyzed across 1,280 elements, which were categorized into six major classes: LINE, SINE, LTR, RNA element, satellite, and transposable element. Among them, 181 belonged to LINE, 60 to SINE, 568 to LTR, 73 to RNA element, 47 to satellite, and 351 to transposable element. Unsupervised clustering based on REP separated cancer and healthy samples (Fig. 2D), although substantial intra-group heterogeneity was observed in both cohorts. Among these 296 elements, the relative representation of satellites and transposable elements was increased in cancer (fold change > 1), LTRs showed little change, and LINEs, SINEs and RNA elements were decreased (Fig. 2E).

### Model performance in the training and validation cohorts

**Fig. 3:**
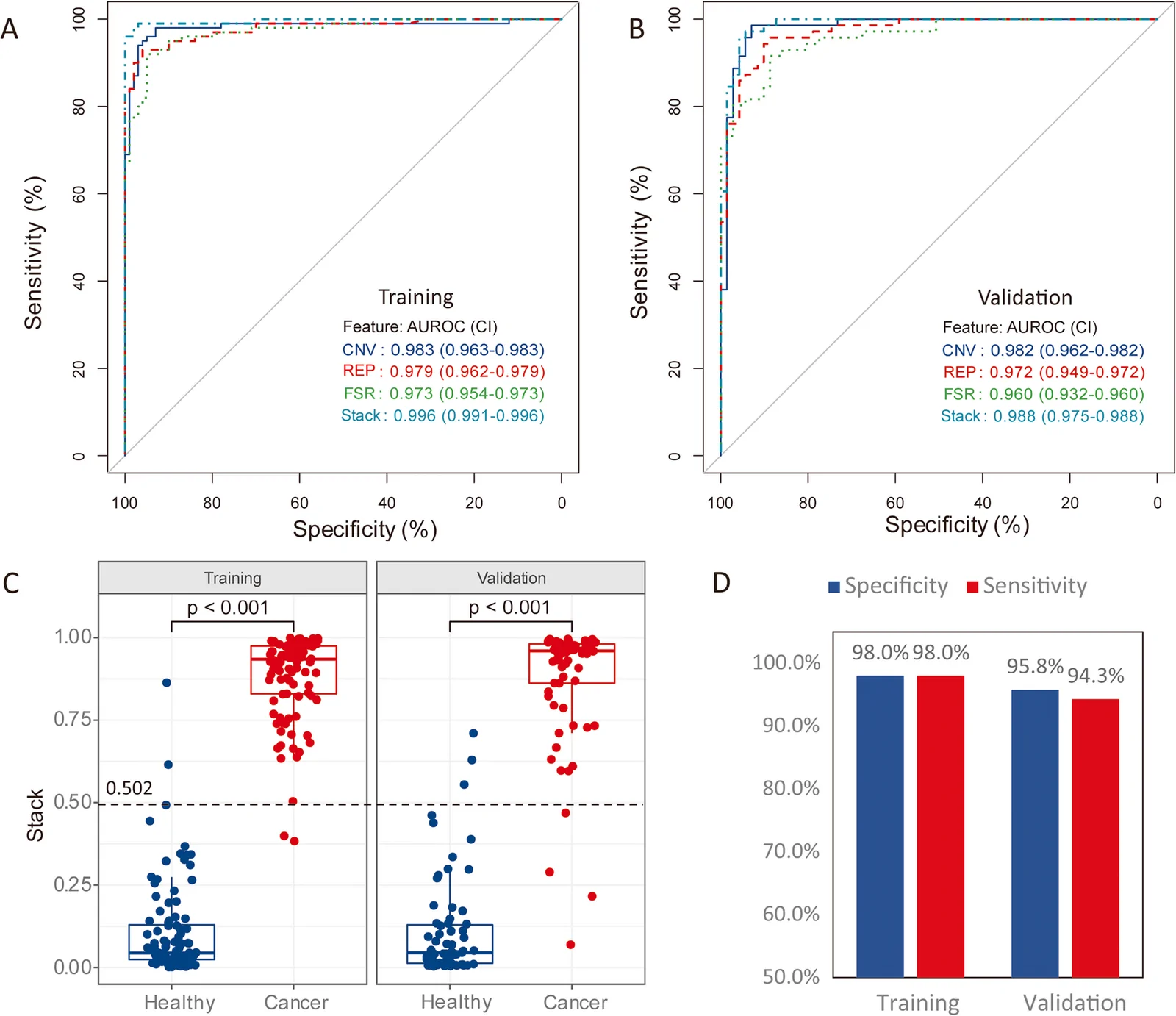
Performance evaluation of the cfDNA fragmentomics-based model. **A** Receiver operating characteristic (ROC) curves of base model (each feature) and stacked model (combined features) in the training cohort. **B** ROC curves of base model and stacked model in the validation cohort. **C** Boxplot of the prediction scores for all samples. **D** Specificity and sensitivity of the stacked model in the training and validation cohorts

Three cfDNA features (FSR, CNV and REP), were used individually and in combination to distinguish oral cancer from healthy samples. In the training cohort, the AUCs for individual cfDNA features were 0.973 (95% CI: 0.954–0.992) for FSR, 0.983 (95% CI: 0.963–1.000) for CNV, and 0.979 (95% CI: 0.962–1.000) for REP, while in the validation cohort, the corresponding AUCs were 0.960 (95% CI: 0.932–0.987), 0.982 (95% CI: 0.962–1.000), and 0.972 (95% CI: 0.949–0.994), respectively. CNV showed the highest individual performance. Integration of all three cfDNA features yielded AUCs of 0.996 (95% CI: 0.991–1.000) in the training cohort and 0.988 (95% CI: 0.975–1.000) in the validation cohort (Fig. 3A and B). The paired DeLong’s test showed that, in the combined cohort, the stacked model achieved a statistically significant improvement in AUC compared with the CNV-only model (*P* = 0.019). When the training and validation cohorts were analyzed separately, the difference approached statistical significance in the training cohort (*P* = 0.051), but was not statistically significant in the validation cohort (*P* = 0.208). To benchmark our assay, we evaluated the performance of the ichorCNA-estimated TF alone. Although the estimated TF was significantly higher in oral cancer patients than in healthy controls (*P* < 0.01; Figure S2A), the absolute values were notably low, with the majority of cancer samples exhibiting a TF below 3%. Consequently, the TF-based classifier achieved only modest diagnostic utility, with AUCs of 0.607 and 0.648 in the training and validation cohorts, respectively (Figure S2B).

The stacked model generated a prediction score (0 to 1) representing the probability of oral cancer. Scores were significantly higher in cancer patients than in healthy controls in both training (*P* < 0.001) and validation cohorts (*P* < 0.001; Fig. 3C). To align with clinical requirements for high diagnostic confidence in oral cancer screening, the decision threshold was determined by prioritizing high specificity (targeted at 98.0%) within the training cohort. This resulted in an optimal cut-off of 0.502, which was subsequently locked and applied unchanged to the independent validation cohort. At this locked threshold, the model achieved 98.0% sensitivity (95% CI: 93.0%–99.4%) and 98.0% specificity (95% CI: 93.0%–99.4%) in the training cohort, and 94.4% sensitivity (95%CI: 86.4%–97.8%) and 95.8% specificity (95% CI: 88.3%–98.6%) in the validation cohort (Fig. 3D). To further compare the performance of individual feature classes, we calculated the sensitivity and specificity of each single-feature model in the independent validation cohort. The CNV-only, REP-only, and FSR-only models achieved specificities of 95.8% (95% CI: 88.3%–98.6%), 95.8% (95% CI: 88.3%–98.6%), and 87.3% (95% CI: 77.6%–93.2%), respectively, with corresponding sensitivities of 94.4% (95% CI: 86.4%–97.8%), 85.9% (95% CI: 76.0%–92.2%), and 88.7% (95% CI: 79.3%–94.2%). The stacked model achieved a sensitivity of 94.4% (95% CI: 86.4%–97.8%) and a specificity of 95.8% (95% CI: 88.3%–98.6%) in the same validation cohort.

### Model performance across cancer subgroups

**Fig. 4:**
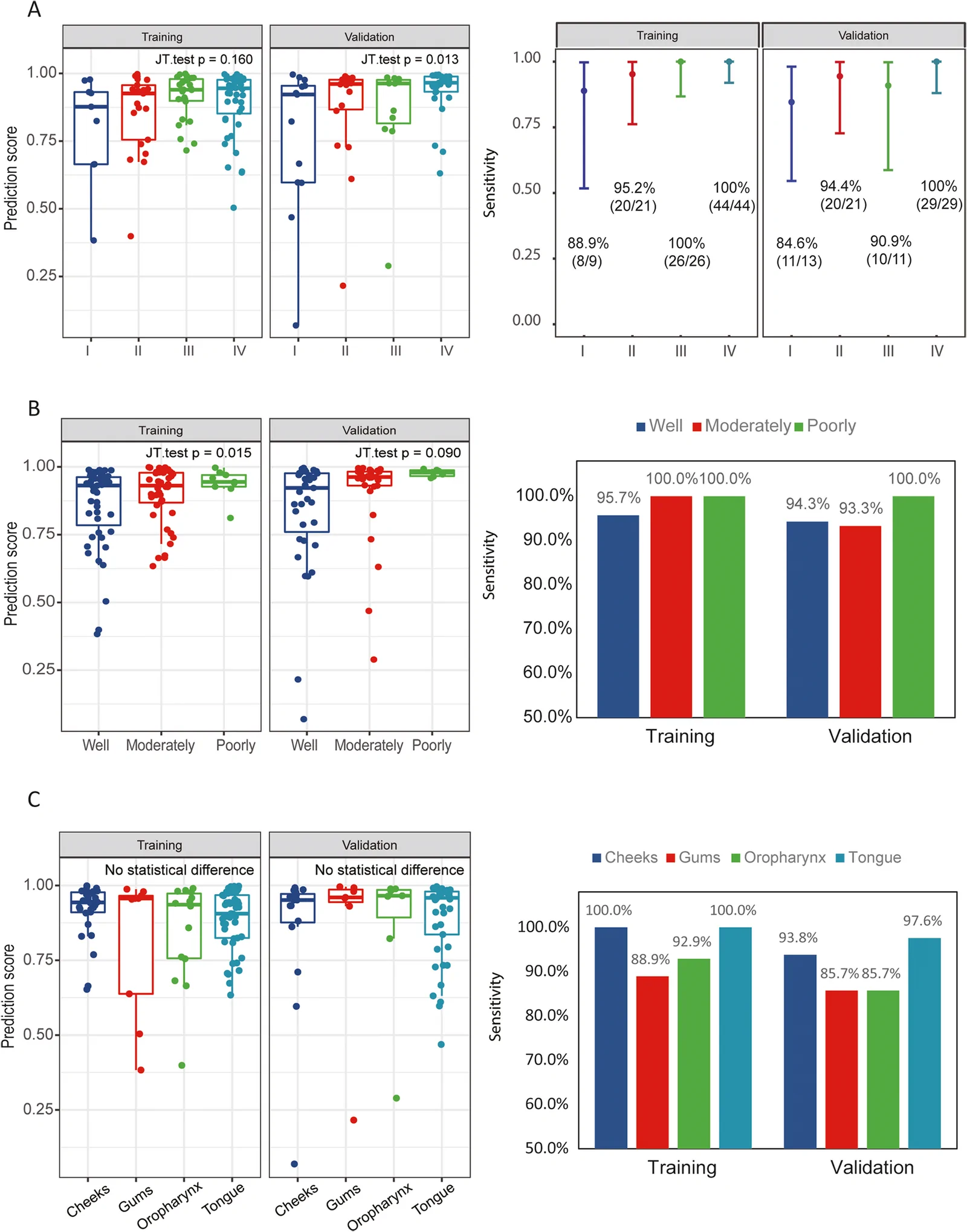
Model performance across clinical subgroups. **A** Boxplots and sensitivity according to clinical stage in the training and validation cohorts. **B** Boxplots and sensitivity according to histological differentiation degree in the training and validation cohorts. **C** Boxplots and sensitivity according to tumor location in the training and validation cohorts

Pathological staging is a cornerstone of oral cancer management and prognosis. We evaluated the model’s predictive scores across clinical stages using the Jonckheere-Terpstra (JT) test (Fig. 4A). While scores showed a numerical upward trend in the training cohort (*P* = 0.160), this association reached statistical significance in the validation cohort (*P* = 0.013). The model showed high sensitivity across all stages (Fig. 4A). For early-stage disease, the sensitivity for stage I cancers was 88.9% (95% CI: 51.8%–99.7%) in training and 84.6% (95% CI: 54.6%–98.1%). In the training cohort, the sensitivities for detecting stage II, III, and IV oral cancers were 95.2% (20/21), 100% (26/26), and 100% (44/44), respectively. These results were comparable in the independent validation cohort, yielding sensitivities of 94.4% (17/18) for stage II, 90.9% (10/11) for stage III, and 100% (29/29) for stage IV disease. Detailed sensitivity values and 95% CIs for all subgroups are provided in Table S4.

To ensure the model’s diagnostic reliability, we further investigated the potential influence of demographic and clinical confounding factors. Predictive scores were found to be independent of sex, with no significant differences observed between male and female participants in either the healthy (*P* = 0.54) or cancer (*P* = 0.60) cohorts (Figure S3A). Similarly, age showed a negligible correlation with prediction scores across both groups (R^2^ = 0.001 for cancer and R^2^ = 0.005 for healthy individuals; Figure S3B). We also evaluated the model’s performance across individual pathological sub-groups (Figure S3C). While prediction scores remained consistently high in cancer patients compared to healthy controls, the subtle numerical increases observed across advancing T stages (JT test, *P* = 0.186 in training and *P* = 0.189 in validation) and N stages (JT test, *P* = 0.290 in training and *P* = 0.233 in validation) did not reach statistical significance. Regarding distant metastasis, while the training cohort showed a nominally higher prediction score in M1 cases (*P* = 0.024), this was not statistically significant in the validation cohort (*P* = 0.80). Given that the M1 subgroup was limited (*n* = 3), these findings regarding metastatic spread should be interpreted with caution. Individual predictive scores and comprehensive clinical metadata for all participants are provided in Tables S2 and S3.

Predictive scores increased with decreasing differentiation grade in both the training (*P* = 0.015) and validation (*P* = 0.09) cohorts (Fig. 4B). In the training cohort, sensitivities were 95.7% (45/47), 100% (44/44), and 100% (9/9) for well, moderately, and poorly differentiated tumors, respectively. In the validation cohort, sensitivities were 94.4% (33/35), 93.3% (28/30), and 100% (6/6), showing a generally increasing trend with decreasing differentiation (Fig. 4B).

Given the diverse anatomical origins of oral cancer, including the cheek, gum, oropharynx, and tongue, we further examined the effects of tumor location on prediction scores. No significant differences in predictive scores were observed across anatomical sites in either the training or validation cohorts (Fig. 4C). High sensitivities were maintained across all sites. Tumors of the cheek and tongue showed particularly robust detection rates, with 100% (26/26) and 100% (40/40) in the training cohort and 93.8% (15/16) and 97.6% (40/41) in the validation cohort. Lower sensitivities were observed for gum and oropharyngeal cancers, likely due to limited sample sizes, with 88.9% (8/9) and 92.9% (13/14) in the training cohort and 85.7% (6/7) for both sites in the validation cohort (Fig. 4C).

## Discussion

Early detection of oral cancer is crucial for improving patient prognosis, yet current screening methods remain invasive or insufficiently sensitive. In this study, we developed and validated a cfDNA fragmentomics-based liquid biopsy assay capable of distinguishing oral cancer from healthy individuals. By integrating three cfDNA-derived features, including FSR, CNV and REP, through a stacked model, our assay achieved AUCs of 0.996 and 0.988 in the training and validation cohorts, respectively. Notably, the assay demonstrated high sensitivity for early-stage (I–II) cancers, suggesting its potential for further evaluation in early oral cancer detection.

The observed cfDNA fragmentomic alterations are consistent with the biological characteristics of malignancy. Cancer-derived cfDNA is typically shorter, a phenomenon attributed to altered chromatin accessibility and nuclease-mediated cleavage patterns during apoptotic and necrotic DNA fragmentation [29, 30]. The elevated short-to-long fragment ratio observed in our study aligns with these mechanistic observations and fragmentomic signatures reported across multiple cancer types [31]. Similarly, CNV analysis revealed frequent gains at 3q and 8q and losses at 3p and 5q, involving key oncogenes (*CCND1* and *MYC*) and tumor suppressors (*CDKN2A* and *PTEN*). These alterations mirror the genomic instability characteristic of oral squamous cell carcinoma and provide biological plausibility for cfDNA-based detection [32, 33]. REP analysis further revealed increased representation of satellite and transposable elements in cancer samples, consistent with widespread epigenetic deregulation and repetitive element reactivation during carcinogenesis, a phenomenon also detectable in cfDNA repeat landscapes [18, 34] .

To place our findings in a broader clinical context, we compared our fragmentomics-based assay with current oral cancer screening modalities reviewed in the recent American Head and Neck Society (AHNS) position statement [35]. Traditional diagnostic standards, such as incisional biopsy, are invasive and may be delayed by clinical lag time. Non-invasive alternatives, including brush cytology and biomarker-based approaches, such as salivary cytokines, exosomal miRNAs, or methylation-based assays, have shown variable diagnostic performance [36, 37]. Notably, the low baseline performance of traditional tumor fraction estimation (ichorCNA AUC ~ 0.61–0.65) confirms the inherent difficulty of the diagnostic task. In the present cohort, our cfDNA fragmentomics-based assay showed promising diagnostic performance using low-depth plasma WGS. However, cross-study comparisons should be interpreted cautiously because of differences in study design, study populations, assay platforms, and performance metrics. The low diagnostic performance of tumor fraction alone in our samples also suggests that integrating multiple cfDNA fragmentomic features may provide additional information beyond conventional tumor fraction estimation. Large multicancer early detection (MCED) studies provide relevant background for cfDNA-based cancer detection assays. The GRAIL/CCGA clinical validation study reported performance for the head and neck cancer subgroup within a targeted methylation-based MCED assay across more than 50 cancer types [9]. These data should be considered cautiously in relation to the present study because of differences in study design, cancer spectrum, assay type, specificity thresholds, sample size, and intended-use population.

The potential added value of the multi-feature stacked model is supported by its incremental improvement over individual feature-specific models, including the CNV-only model. In the paired DeLong’s test, the AUC difference between the stacked model and the CNV-only model reached statistical significance in the combined cohort, whereas the difference was not statistically significant in the independent validation cohort. This may, at least in part, reflect the smaller sample sizes and limited statistical power of the individual cohorts, particularly for detecting modest differences between two models with similarly high performance. From a biological perspective, integration of CNV, FSR, and REP remains plausible because these features capture different aspects of cfDNA alterations. Such integration may reduce reliance on any single feature class and may contribute to more stable classification.

Our model showed generally high sensitivity across several clinical subgroups, including different disease stages, histological grades, and anatomical subsites. However, subgroup-specific estimates should be interpreted cautiously because of limited sample sizes in some categories. Predictive scores showed no clear association with age or sex, suggesting that model performance was not substantially influenced by these demographic variables in this cohort.

Several limitations should be acknowledged. First, the cancer participants in this study were clinically detected cases rather than cancers identified through prospective screening. Although early-stage oral cancers may be painless, all cancer cases in this cohort had visible or suspicious oral mucosal lesions that prompted biopsy. Thus, the model’s performance may not directly generalize to a true asymptomatic screening population, where tumor burden and cfDNA signals may be lower. Second, the study was conducted at a single center, and external validation in multi-center cohorts is warranted to assess generalizability. Third, sample size and distribution across subgroups were uneven, which may affect subgroup-specific estimates of sensitivity. Furthermore, our findings regarding distant metastasis (M stage) must be considered exploratory, as the number of M1 cases in our cohort was extremely limited (*n* = 3). While the model showed a nominal increase in scores for metastatic cases in the training set, these results should be interpreted with caution. Future studies with larger cohorts of advanced-stage patients are needed to better evaluate the model’s performance across metastatic subgroups.

In conclusion, we developed a cfDNA fragmentomics-based liquid biopsy assay for noninvasive oral cancer detection. By leveraging genome-wide fragmentation, CNV, and repetitive element patterns, this assay showed promising performance in distinguishing oral cancer from healthy individuals, including early-stage disease. These findings support further evaluation of cfDNA fragmentomics as a potential approach for early oral cancer detection.

## Supplementary Information

Supplementary Material 1: Figure S1. Distribution of sequencing depth after down-sampling. Box-and-whisker plots showing the sequencing coverage (depth) for oral cancer patients and healthy controls within the training and validation cohorts. Each point represents an individual sample.

Supplementary Material 2.

Supplementary Material 3.

Supplementary Material 4.

Supplementary Material 5: Figure S2. Evaluation of diagnostic performance using ichorCNA-estimated tumor fraction. (A) Comparison of tumor fractions between oral cancer patients and healthy individuals (**, P < 0.01, Wilcoxon rank-sum test). (B) Receiver operating characteristic (ROC) curves and AUC values for the training and validation cohorts using tumor fraction as the classifier.

Supplementary Material 6.

Supplementary Material 7: Figure S3. Associations between prediction scores and demographic or clinical characteristics. (A) Association between sex and prediction scores in healthy individuals and patients with oral cancer in the training cohort. Boxplots showing the distribution of prediction scores by sex in the healthy and oral cancer groups. (B) Correlation between age and prediction scores in healthy individuals and patients with oral cancer in the training cohort. (C) Prediction scores across T, N, and M stages in the training and validation cohorts. Boxplots showing the distribution of prediction scores according to T stage (tumor size), N stage (lymph node status), and M stage (metastasis) in both the training and validation cohorts.

## Data Availability

The raw sequencing data generated during this study have been deposited in the Genome Sequence Archive for Human (GSA-Human) under the accession number HRA017253. Any additional information of this study will be available from the corresponding author upon reasonable request.

## Abbreviations

OSCC: Oral squamous cell carcinoma
cfDNA: Cell-free DNA
cfRNA: Cell-free RNA
ctDNA: Circulating tumor DNA
CNV: Copy number variation
FSR: Fragment size ratio
REP: Repetitive element profiles
WGS: Whole-genome sequencing
AUC: Area under the curve
PON: Panel of normals
MAPQ: Mapping quality
GLM: Generalized linear model
XGBoost: Extreme gradient boosting
GBM: Gradient Boosting Machines
DRF: Deep Random Forest
XRT: eXtreme Randomized Trees
DL: Deep Learning
LINEs: Long interspersed nuclear elements
SINEs: Short interspersed nuclear elements
LTRs: Long terminal repeats
