# Supplementary material for: A cfDNA fragmentomics-based liquid biopsy assay for early detection of oral cancer
Source: BMC Cancer. 2026 Aug 15;26:1395. doi: 10.1186/s12885-026-16606-x (PMC13753169; doi:10.1186/s12885-026-16606-x)
Supplement: Supplementary file 1 [file 12885_2026_16606_MOESM1_ESM.pdf]

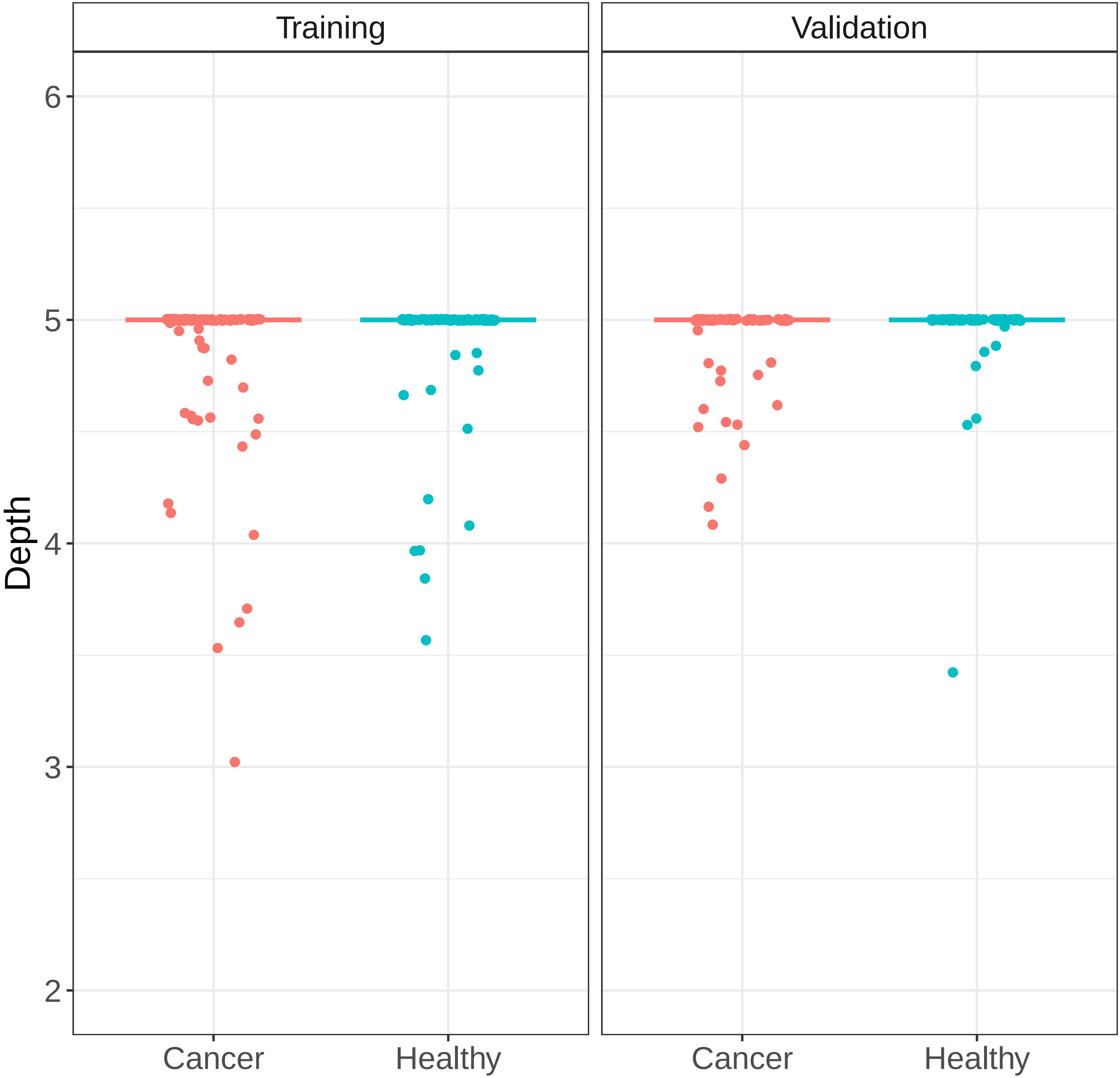
Training
Validation
Depth
6
5
4
3
2
Cancer
Healthy
Cancer
Healthy
