# Supplementary material for: A cfDNA fragmentomics-based liquid biopsy assay for early detection of oral cancer
Source: BMC Cancer. 2026 Aug 15;26:1395. doi: 10.1186/s12885-026-16606-x (PMC13753169; doi:10.1186/s12885-026-16606-x)
Supplement: Supplementary file 2 [file 12885_2026_16606_MOESM5_ESM.pdf]

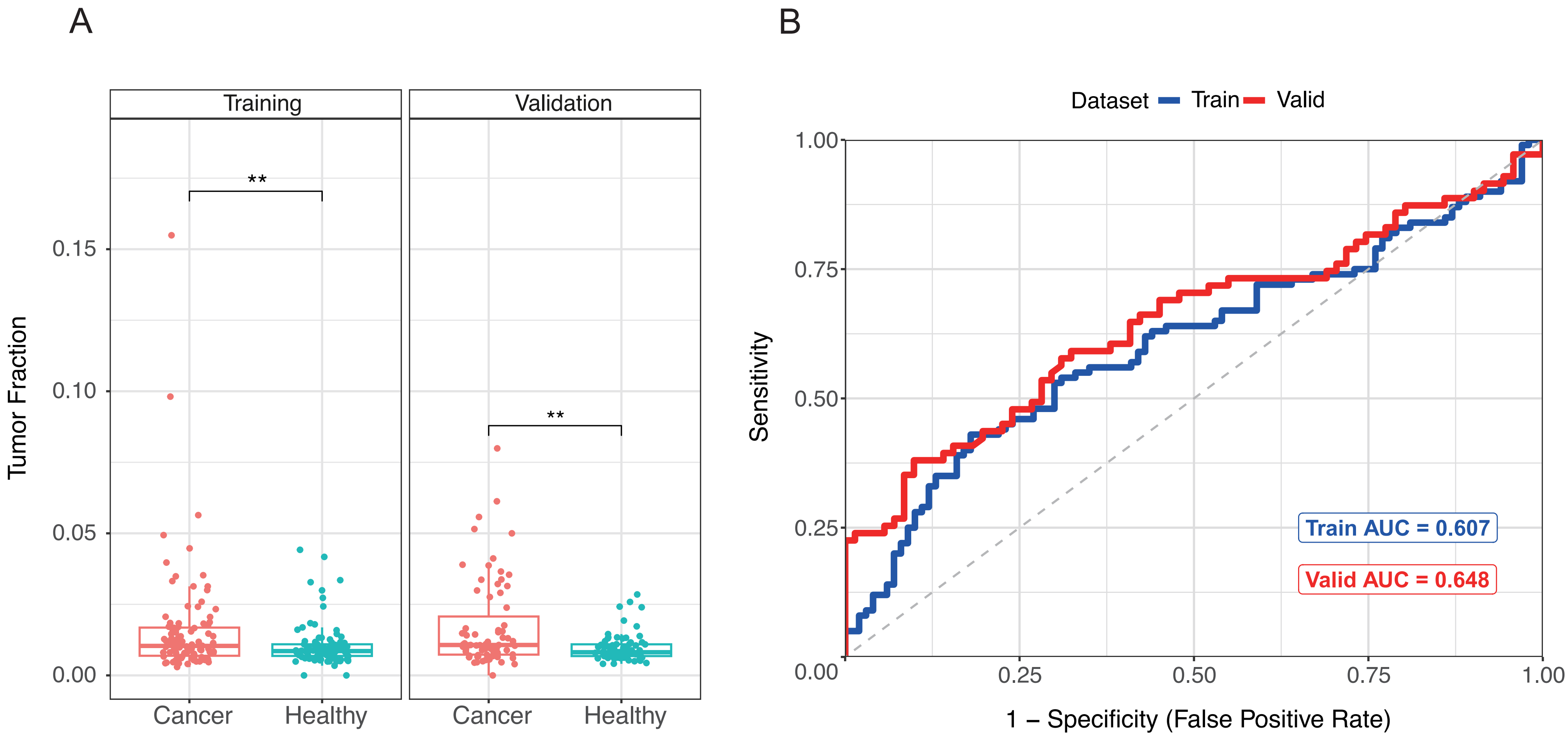
A
B
Training
Validation
Tumor Fraction
0.15
0.10
0.05
0.00
**
Cancer
Healthy
Dataset
Train
Valid
Sensitivity
1.00
0.75
0.50
0.25
1 – Specificity (False Positive Rate)
Train AUC = 0.607
Valid AUC = 0.648
