# Supplementary material for: A cfDNA fragmentomics-based liquid biopsy assay for early detection of oral cancer
Source: BMC Cancer. 2026 Aug 15;26:1395. doi: 10.1186/s12885-026-16606-x (PMC13753169; doi:10.1186/s12885-026-16606-x)
Supplement: Supplementary file 3 [file 12885_2026_16606_MOESM4_ESM.docx]

**Table S1**. Summary of hyperparameter optimization and fixed configurations for the machine learning base learners.

| **Algorithm** | **Hyperparameters Optimized** | **Fixed Parameters/ Default Configurations** |
| --- | --- | --- |
| GLM | alpha = {0.0, 0.2, 0.4, 0.6, 0.8, 1.0} | lambda_search=TRUE; all other GLM settings at default |
| XGBoost | booster, col_sample_rate, col_sample_rate_per_tree, max_depth, min_rows, reg_alpha, reg_lambda, sample_rate | ntrees=10000 with early stopping; all other XGBoost settings at default |
| GBM | col_sample_rate, col_sample_rate_per_tree, max_depth, min_rows, min_split_improvement, sample_rate | learn_rate=0.1, ntrees=10000 with early stopping; all other GBM settings at default |
| H2O deep learning | epsilon, hidden, hidden_dropout_ratios, input_dropout_ratio, rho | activation=RectifierWithDropout, epochs=10000 with early stopping, adaptive_rate=TRUE; all other settings at default |
| DRF / XRT | Not grid-searched in current AutoML | Default settings |

Note: All parameters were optimized using the H2O AutoML (version 3.42) native random grid search procedure.
